# Effects of steroidal saponins extract from *Ophiopogon japonicus* root ameliorates doxorubicin-induced chronic heart failure by inhibiting oxidative stress and inflammatory response

**DOI:** 10.1080/13880209.2019.1577467

**Published:** 2019-03-12

**Authors:** Zhongwei Wu, Xuekai Zhao, Akira Miyamoto, Shengji Zhao, Chaoquan Liu, Weimin Zheng, HongTao Wang

**Affiliations:** a Department of Cardiology, Hainan Western Central Hospital, Danzhou, China;; b Department of Cardiac Surgery, Zibo Central Hospital, Zibo, China;; c Department of Rehabilitation, Kobe International University, Kobe, Japan;; d Department of Cardiology, The Second Affiliated Hospital of Xi’an JiaoTong University, Xian, China

**Keywords:** Cardiovascular diseases, antioxidant effect, cardiotoxicity, inflammatory cytokines

## Abstract

**Context:** Ophiopogonis Radix, the root of *Ophiopogon japonicus* (Thunb.) Ker-Gawl (Liliaceae), is a Traditional Chinese Medicine, which has been investigated to possess effective treatment of cardiovascular diseases.

**Objective:** This study evaluates the cardioprotective effects of steroidal saponins extract from *Ophiopogon japonicus* (SOJ) root against doxorubicin-induced chronic heart failure (CHF) through the amelioration of oxidative stress and inflammation.

**Materials and methods:** A Sprague-Dawley rat model of CHF was established by intraperitoneally injected with DOX. All rats were randomly divided into four groups: Control group, CHF group, CHF + SOJ (100 mg/kg) treatment group, SOJ (100 mg/kg) treatment group (*n* = 8/group). After six weeks administration, biometric and echocardiography were measured. The levels of biochemical parameters were measured using commercial kits.

**Results:** The values of LVESP, +dP/dtmax, –dP/dtmax, EF and FS increased to 116.20 ± 1.68 mmHg, 2978.71 ± 168.26 mmHg/s, 3452.61 ± 286.09 mmHg/s, 68.26 ± 5.28% and 31.97 ± 3.79%, respectively; the values of LVEDP, LVESD and LVEDD decreased to 8.85 ± 0.84 mmHg, 8.39 ± 0.45 mm and 12.36 ± 0.87 mm in CHF + SOJ group. In addition, the levels of IL-6, TNF-α and IL-1β decreased to 154.41 ± 7.72 pg/mg protein, 110.02 ± 6.96 pg/mg protein and 39.39 ± 5.27 pg/mg protein, respectively; the relative activity of p38 MAPK decreased to 2.60 ± 0.40 in CHF + SOJ group. Furthermore, the activities of SOD, CAT and GSH-Px increased to 268.77 ± 6.20 U/mg protein, 13.68 ± 0.68 U/mg protein and 316.90 ± 8.08 µmol/mg protein, and the content of MDA decreased to 4.03 ± 0.43 nmol/mg protein in CHF + SOJ group.

**Conclusions:** SOJ exerts the cardioprotective effect against DOX-induced CHF through suppressing inflammatory and oxidative stress. These results provide evidence that SOJ might be an effective treatment for CHF.

## Introduction

Chronic heart failure (CHF) is one of the most common chronic cardiovascular diseases worldwide; it often causes high morbidity and mortality in clinics. When hearts suffer stress, CHF is characterized by a progressive decrease in cardiac output, as a result of a functional cardiac disorder (Wong et al. 2016). Doxorubicin (DOX), an anthracycline antibiotic drug which has been widely used in the treatment of solid tumours, malignant lymphoma and acute leukaemia (Minotti et al. 2004). However, its clinical utility is restricted partly due to its dose-dependent cardiotoxicity, and the high incidence of severe heart failure (Mazevet et al. 2013). Once it happens, DOX-induced cardiotoxicity is irreversible, consequently progressing to the development of CHF, dilated cardiomyopathy and left ventricular dysfunction (Lipshultz et al. 2012). The mortality rates of CHF incidence often reach up to 20%. Although the underlying molecular mechanisms of DOX-induced cardiomyopathy are multifactorial and have not yet been fully clarified, but oxidative stress, inflammatory response, apoptosis and mitochondrial dysfunction are involved (Childs et al. 2002; Briasoulis et al. 2016). Among these, the overproduction of reactive oxygen species production in cardiomyocyte mitochondria contributes mainly to cardiac damage and dysfunction observed after DOX treatment (Octavia et al. 2012). Accumulated reactive oxygen species can overwhelm the capacity of cardiac antioxidant defence systems and facilitate autophagy and apoptosis in cardiomyocytes, which lead to the development of DOX-induced CHF (Ma et al. 2013). In addition, the pathogenesis and development of CHF also have attributed to chronic pro-inflammatory cytokines, such as tumour necrosis factor-α (TNF-α), interleukin-1β (IL-1β) and interleukin-6 (IL-6), which contribute to endothelial dysfunction, myocardial remodelling and vascular damage (Nian et al. 2004). Therefore, reducing inflammatory response and oxidative stress after CHF serves as a therapeutic method for the treatment of CHF.

Ophiopogonis Radix, the root of *Ophiopogon japonicus* (Thunb.) Ker-Gawl (Liliaceae) is an evergreen perennial widely used and distributed in East Asia, especially in China. *Ophiopogon japonicus* is often used in compound prescriptions, such as YiQiFuMai injection, Sheng Mai Yin and Xuanmai granule. Considerable clinical evidence has showed that Sheng Mai Yin can be used for treatment of myocardial infarction, coronary heart disease and shock (Zhang et al. 2018). Extensive researches have revealed that *Ophiopogon japonicus* has beneficial effects on the cardiovascular diseases through various mechanisms, such as antioxidation, antiarrhythmia, improving microcirculation, etc. (Ichikawa et al. 2003; Kou et al. 2006). Phytochemical research has found that steroidal saponins are one of the main characteristic constituents of *Ophiopogon japonicus*. This group of compounds exhibits therapeutic effects against diabetes, chronic inflammation, oxidative stress and cardiovascular diseases (Chen et al. 2016). However, it is unknown whether saponins-rich extract of *Ophiopogon japonicus* (SOJ) is able to protect cardiomyocytes from DOX-induced CHF via its anti-inflammatory and antioxidative effects.

In the present study, we investigated the cardioprotective effect of SOJ, an extract proven to possess anti-inflammatory and antioxidant effects in DOX-induced CHF. In addition, the HPLC-ELSD modes were used to characterize the bioactive components that responsible of its cardioprotective effect.

## Materials and methods

### Chemicals and drugs

Standards of ophiopogonin D (≥98%), ophiopogonin D′ (≥98%) and ophiopogonin B (≥98%) and DOX were purchased from Sigma-Aldrich (St. Louis, MO). HPLC grade acetonitrile was also purchased from Sigma-Aldrich (St. Louis, MO). The roots of *Ophiopogon japonicus* were obtained from Tongrentang Chinese Medicine (Beijing, China) and raw medicinal materials were harvested in April 2018, and identified by Dr Xuekai Zhao according to the Pharmacopeia of the People’s Republic of China. A voucher specimen of *Ophiopogon japonicus* (No. 201802) was deposited at the College of Pharmacy of Xi’an JiaoTong University. All other chemical reagents with analytical grade were purchased from Sinopharm Chemical Reagent Co., Ltd (Shanghai, China).

### Preparation of SOJ

The dried roots of *Ophiopogon japonicus* were crushed to powder (80 mesh sizes) and extracted three times with 80% ethanol for 1 h under reflux condition. After that, the extract was filtered and concentrated under vacuum at 50 °C to obtain the crude *Ophiopogon japonicus* extracts. Then, the crude extracts were added into the AB-8 resin for 12 h, and the AB-8 resin column was washed with ultrapure water to remove the polar impurities, and then eluted by 70% ethanol at a flow rate of 1.5 mL/min. The eluate was collected, and then lyophilized with freeze-dryer (Zhou et al. 2017). Finally, the contents of total steroidal saponins were assayed using a diosgenin as reference by UV spectrophotometry.

### Chromatogram analysis of SOJ by HPLC-ELSD

The chromatogram analysis was carried out on a Waters 600E system equipped with Luna C18 (2) column (250 mm × 4.6 mm, 5 μm), Waters evaporative light-scattering detector (ELSD), and a quaternary pumps. The column temperature was 30 °C and flow was set 0.8 mL/min. The analysis of mobile phase consisted of water (A) and acetonitrile (B) was applied as follows: 80% (A) to 40% (A) for 40 min, and 40% (A) held for 10 min. The ELSD settings as follows: nitrogen flow rate (3.0 L/min), and drift tube temperature (100 °C).

### Animals

The experiments were performed using male Sprague-Dawley rats (10–12 week-old) with a body weight range of 240–280 g. All rats were purchased from the Experimental Animal Center of Hainan Province and bred in polypropylene cages under conditions of 22–26 °C, with a 12 h light/dark cycles, and free access to drinking water and standard rodent chow. All animal experimental procedures were approved by the Ethics Committee in accordance with the guidelines for the Care and Use of Laboratory Animals (NIH Publications No. 85-23, revised 1996).

### Experimental design and protocol

The SD rats were randomly assigned into four groups (*n* = 8/group): control, CHF, CHF + SOJ and SOJ groups. Rats in the control group were received water every day orally via gavage, and saline intraperitoneally every second day; rats in CHF group were received water orally, and intraperitoneally injected with DOX at the dose of 2.5 mg/kg body weight every second day for six times (the cumulative dose was 15 mg/kg) (Namdari and Eatemadi 2017); rats in the SOJ group were received SOJ at a dose of 100 mg/kg every day via gavage; rats in the CHF + SOJ group were received SOJ every day orally and intraperitoneally injected with DOX every other day at the above mentioned dose. The rats were treated for a total of 6 weeks.

### Evaluation of cardiac injury markers

After 24 h of the last dose treatment, blood samples were collected from the retro-orbital plexus. The cardiac damage markers of animal serum were measured using commercial kits, including creatine kinase-MB (CK-MB), aspartate aminotransferase (AST) and lactate dehydrogenase (LDH). These commercial kits were purchased from Nanjing Jiancheng Bioengineering (Nanjing, China).

### Hemodynamic measurements

After 6 weeks of administration with SOJ, all animals were anesthetized with intraperitoneal injections of 100 mg/kg thiobutabarbital. A micro catheter with a pressure transducer was inserted into right carotid arteries and advanced to left ventricular cavity for measurement of hemodynamic parameters. After 5 min of accommodation, left ventricular end-diastolic pressure (LVEDP) and left ventricular end-systolic pressure (LVESP) were recorded. Meanwhile, the hemodynamic parameters of the left ventricular pressure (±dP/dtmax) were also assayed. The measurements of hemodynamic parameters were performed using an AD Instruments Power Lab System (ML750, Castle Hill, Australia).

### Echocardiographic measurements

After the measurement of hemodynamic parameters, all animals were subjected to transthoracic M-mode echocardiography using a high-resolution system (ACUSON Sequoia 512, Siemens, Munich, Germany). The left ventricular end systolic diameter (LVESD) and the left ventricular end diastolic diameter (LVEDD) were assayed by using a 5–10 MHz broadband transducer. The left ventricular ejection fraction (EF) and left ventricular fractional shortening (FS) were calculated as follows:

$$\text{EF}=\left({\text{LVEDD}}^{3}-{\text{LVESD}}^{3}\right){/\text{LVEDD}}^{3}\text{;}\;\text{FS}=\left(\text{LVEDD}-\text{LVESD}\right)/\text{LVEDD}$$

### Measurement of oxidative stress and inflammatory cytokines

At the end of experiment, the rats were euthanized by pentobarbital overdose. The hearts were immediately isolated, weighted and homogenized with ice-cold physiological saline. Thereafter, the homogenate was centrifuged at 5000 rpm for 10 min and the supernatant was collected for further analysis biochemical parameters. Evaluation of the levels of malondialdehyde (MDA), glutathione peroxidase (GSH-Px), superoxide dismutase (SOD), catalase (CAT), total protein, IL-6, TNF-α and IL-1β in myocardial homogenates were carried out using commercial kits according to the manufacturer’s guidelines (Nanjing Jiancheng Bioengineering Institute, Nanjing, China). The measurement of p38 MAPK activity was carried out using a p38 MAPK activity assay kit (Cell Signaling Technology, Beverly, MA) as described in previous research (Tao et al. 2006).

### Statistical analysis

All data were obtained from all groups and expressed as mean ± standard deviation (SD). Statistical tests were performed with the SPSS 22.0 software (SPSS Inc., Chicago, IL). Comparisons were carried out by one-way ANOVA for the different groups, followed by the Tukey comparison test. *p* < 0.05 was considered statistically significant.

## Results

### HPLC-ELSD profiles of SOJ extract

**Figure 1.**
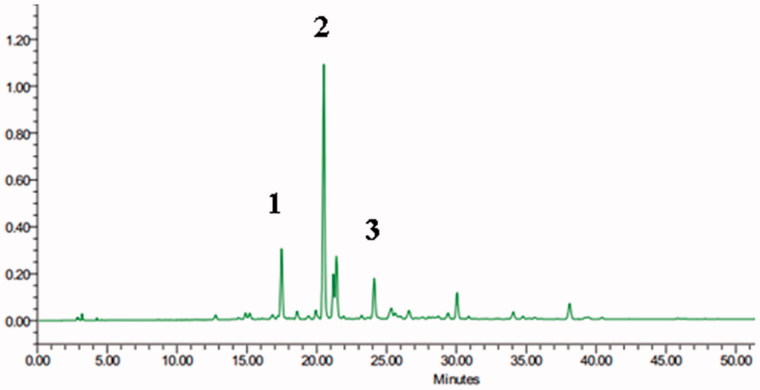
The HPLC-ELSD chromatogram of saponins-rich fraction of *Ophiopogon japonicus* (SOJ). Peak: 1, ophiopogonin B; 2, ophiopogonin D; 3, ophiopogonin D′.

As displayed in Figure 1, HPLC-ELSD analysis shows the major bioactive constituents with moderate polarity during 0–40 min retention time. The three observed steroidal saponins were assigned as ophiopogonin B (1), ophiopogonin D (2) and ophiopogonin D′ (3), respectively, by comparing the retention times with the reference in HPLC chromatograms.

### SOJ improved cardiac function in DOX-induced CHF rats

**Figure 2.**
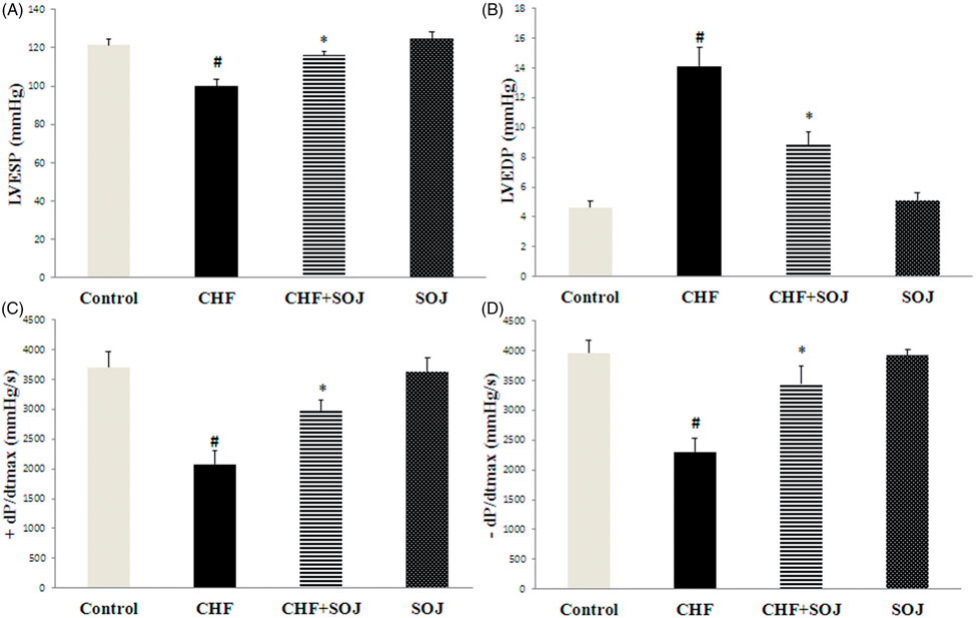
Comparison of the values of hemodynamic parameters after treatment with SOJ between experimental groups. Data are expressed as mean ± SD, #*p* < 0.05 versus control group, *p < 0.05 versus CHF group. LV ± dP/dtmax: the instantaneous first derivation of left ventricle pressure; LVESP: left ventricular systolic pressure; LVEDP: left ventricular end diastolic pressure; SOJ: saponins-rich fraction of *Ophiopogon japonicus*.

As shown in Figure 2, the LVESP and ± dP/dtmax showed a reduction in the CHF group (17.77%, 44.05% and 41.93%, respectively; *p* < 0.05), whereas LVEDP was increased in the CHF group in comparison with the control group (203.95%, *p* < 0.05). However, an obvious improvement of hemodynamic parameters was observed after 6 weeks of treatment with SOJ when compared with the CHF group (16.40%, 43.56%, 50.00% and 37.29%, respectively; *p* < 0.05).

**Figure 3.**
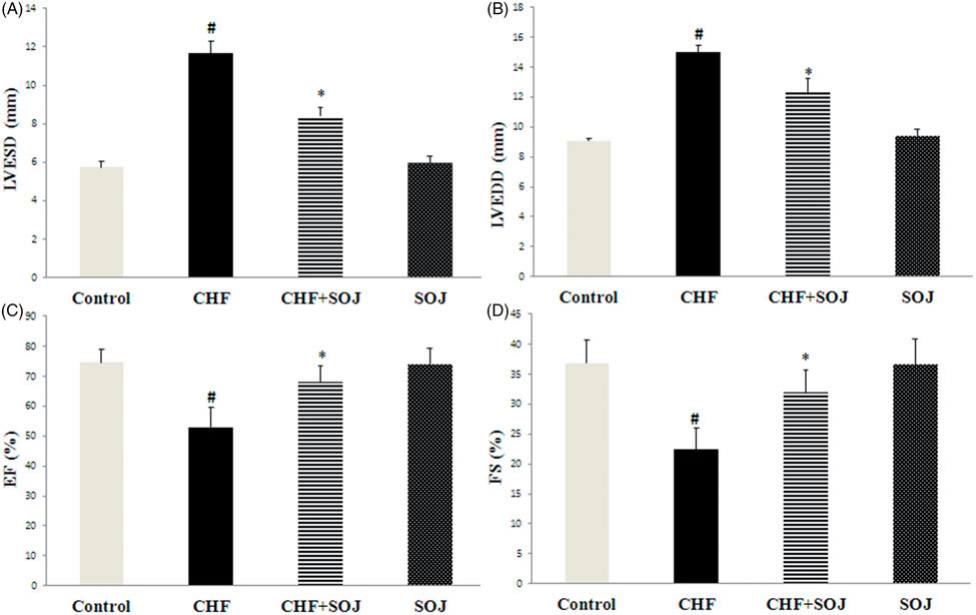
Comparison of the values of echocardiographic parameters after treatment with SOJ between experimental groups. Data are expressed as mean ± SD, #*p* < 0.05 versus control group, *p < 0.05 versus CHF group. LVESD: left ventricular end-systolic diameter; LVEDD: left ventricular end-diastolic diameter; EF: left ventricular ejection fraction; FS: left ventricular fractional shortening; SOJ: saponins-rich fraction of *Ophiopogon japonicus*.

As shown in Figure 3, the LVESD and LVEDD were obviously increased (103.58% and 65.60%, respectively; *p* < 0.05), and the values of EF and FS were significantly decreased in the CHF group when compared with the control group (28.91% and 39.20%, respectively; *p* < 0.05). This showed that the CHF model was established successfully. As expected, treatment with SOJ for 6 weeks obviously decreased the values of LVESD and LVEDD (27.94% and 17.65%, respectively; *p* < 0.05), and obviously improved EF and FS in comparison with those in the CHF group (28.68% and 42.56%, respectively; *p* < 0.05). These results implied that treatment with SOJ improved cardiac function in DOX-induced CHF rats.

### SOJ prevented DOX-induced cardiac toxicity

**Figure 4.**
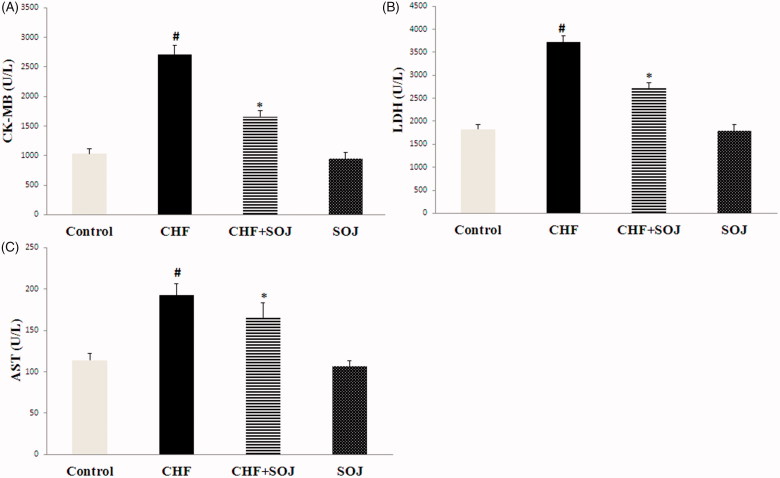
SOJ prevention from DOX-induced cardiac injury *in vivo*. Data are expressed as mean ± SD, #*p* < 0.05 versus control group, *p < 0.05 versus CHF group. CK-MB: creatine kinase-MB; AST: aspartate aminotransferase; LDH: lactate dehydrogenase.

Changes in serum myocardial enzyme (CK-MB, LDH and AST) were measured at 6 weeks of the treatment to assess the extent of myocardial damage. As shown in Figure 4, the levels of serum CK-MB, LDH and AST obviously increased in CHF group when compared with the control group (162.79%, 103.44% and 68.67%, respectively; *p* < 0.05). However, SOJ treatment obviously decreased these serum myocardial enzyme levels compared with the CHF group (38.86%, 26.84% and 13.68%, respectively; *p* < 0.05).

### SOJ treatment ameliorated cardiac oxidative stress

**Figure 5.**
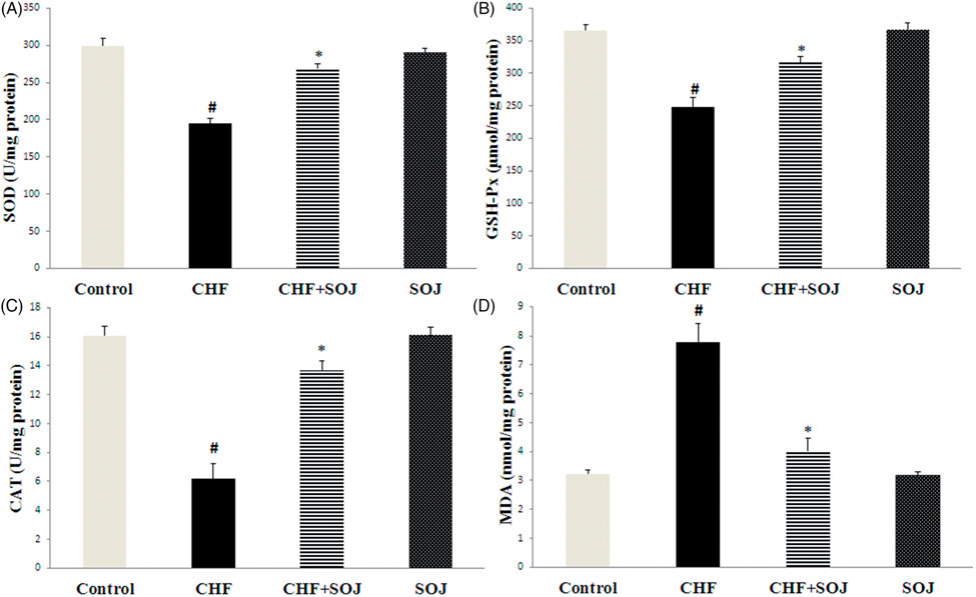
SOJ treatment ameliorated cardiac oxidative stress in CHF rats. At six weeks of treatment, the SOD (A), GSH-Px (B), CAT (C) and MDA (D) levels of the heart tissue were assayed by kits. Data are expressed as mean ± SD, #*p* < 0.05 versus control group, *p < 0.05 versus CHF group.

The activities of SOD, GSH-Px, CAT and the content of MDA were measured to investigate the effect of DOX-induced oxidative stress in heart tissues. As displayed in Figure 5, the activities of SOD, GSH-Px, and CAT were obviously decreased in heart tissues of CHF group compared with the control group (34.97%, 32.16% and 61.61%, respectively; *p* < 0.05), and the content of MDA was obviously increased in CHF group compared with the control group (141.09%, *p* < 0.05). However, SOJ treatment reduced the content of MDA and increased the activities of antioxidant enzymes when compared with the CHF group (48.16%, 37.98%, 27.59% and 121.59%, respectively; *p* < 0.05). These results implied that SOJ treatment suppressed oxidative stress in DOX-induced CHF rats.

### SOJ treatment decreased the DOX-induced inflammatory response

**Figure 6.**
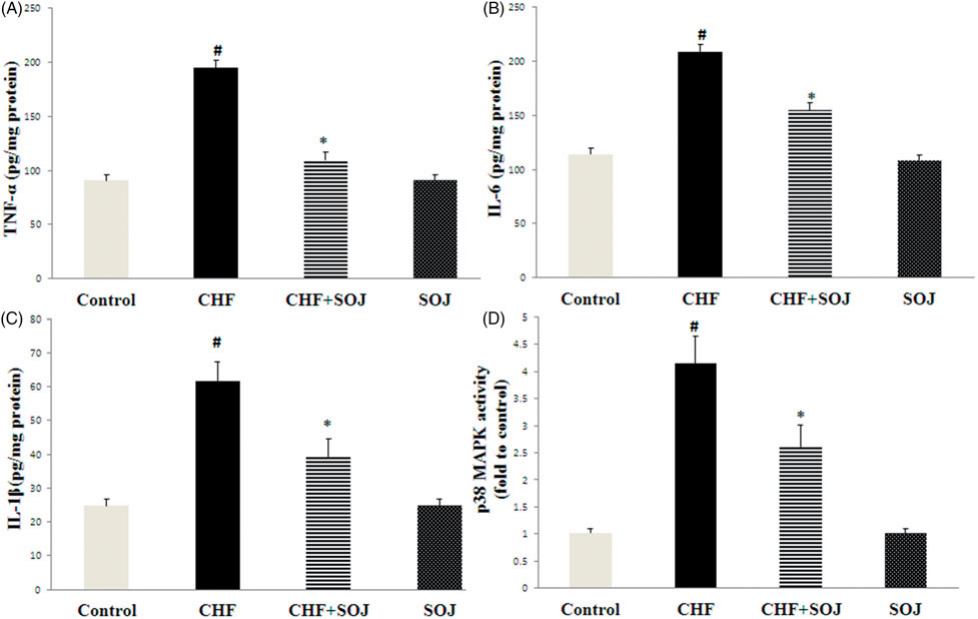
SOJ treatment reduced inflammatory response and p38 MAPK activation in CHF rats. Data are expressed as mean ± SD, #*p* < 0.05 versus control group, *p < 0.05 versus CHF group.

The activity of p38 MAPK was measured to demonstrate whether p38 MAPK signalling pathway participates in the cardioprotective effect of SOJ against DOX-induced CHF. As shown in Figure 6, DOX-induced a significant increase in p38 MAPK activity (307.24%, *p* < 0.05). However, SOJ treatment obviously declined p38 MAPK activity induced by DOX (37.16%, *p* < 0.05). Additionally, the levels of the inflammatory cytokines IL-6, TNF-α and IL-1β in heart tissues were higher in the CHF group compared with the control group (83.29%, 114.69% and 149.07%, respectively; *p* < 0.05). However, SOJ treatment obviously decreased these inflammatory cytokine levels compared with the CHF group (25.96%, 43.51% and 36.09%, respectively; *p* < 0.05).

## Discussion

DOX is a typical anthracycline drugs, which is efficacious against various tumours. However, the clinical application of DOX is limited by its adverse effects that include cardiac toxicity and congestive hearts failure (Zhang et al. 2009). In this regard, searching a novel therapeutic schedule is urgently required. Based on the fact that oxidative stress, excess free radical generation and proinflammatory cytokines are the major triggers in DOX-induced cardiac toxicity (Chatterjee et al. 2010; Briasoulis et al. 2016), treatment strategies have focused in administering natural compounds that alleviate inflammatory response and improve the antioxidant defences against DOX-induced cardiac toxicity. The results of the present research show that DOX treatment result in heart failure and that SOJ exerts cardioprotection in DOX-induced CHF rats. Our results also exhibit that cardioprotection offered by SOJ might be related to its release of proinflammatory cytokines and amelioration of oxidative stress. As far as we know, this research appears to be the first to investigate the effects of SOJ in rat model of CHF.

In this research, DOX-induced CHF model was successfully established through intraperitoneally injected with DOX at the dose of 2.5 mg/kg body weight every second day for six times (the cumulative dose was 15 mg/kg). The underlying molecular mechanism of DOX-induced cardiotoxicity seems to be multifactorial. However, enhanced cardiac inflammatory cytokines and oxidative stress are the major contributors. Our research exhibited that hemodynamic parameters including LV ± dP/dtmax and LVESP were obviously lower, and LVEDP was significantly higher in CHF group than these in control group. Moreover, echocardiographic results indicated that LVESD and LVEDD obviously increased and cardiac function including EF and FS significantly decreased. We also observed a progressive increase in the serum levels of cardiac damage markers (AST, LDH and CK-MB), along with an obvious increase in myocardial oxidative stress and inflammation. These results are similar to those observed previously with experimental heart failure (Petroni et al. 2017; Zilinyi et al. 2018).

Accumulating evidence indicates that oxidative stress can be induced by DOX and results in the production of oxidant, which eventually lead to cardiac tissue oxidative damage, and the previous research has shown that mild hypothermia exerts a possible protective role to reduce the impact of DOX-induced cardiomyopathy by suppressing oxidative stress (L'Ecuyer et al. 2012). Therefore, the extent of oxidative stress was measured by assaying the activities of antioxidant enzymes (SOD, GSH-Px and CAT) and the content of MDA (Rashikh et al. 2012). The present research showed an obvious increase in MDA levels, accompanied by an obvious decrease in the activities of SOD, GSH-Px and CAT in the cardiac tissue of DOX-induced CHF rats. However, SOJ treatment not only alleviated DOX-induced cardiac dysfunction, but also increased the activities of antioxidant enzymes and decreased the content of MDA. These results imply that SOJ may exert cardioprotective effects by alleviating DOX-induced oxidative stress.

In our research, inflammatory cytokines were measured in the CHF model as inflammation is another major factor involved in the development of CHF, and the alleviation of inflammatory cytokines may improve cardiac function (Aukrust et al. 2004). In addition, the activation of p38 MAPK also plays a vital role in DOX-induced cardiac damage (Rose et al. 2010). Moreover, a wide body of evidence has showed that the development of p38 MAPK inhibitors is a potential therapy in the treatment of cardiovascular disease (Clark et al. 2007). Previous study has exhibited that suppression of p38α activation may exert cardioprotection and reduce cardiac damage (Saurin et al. 2000). It has been proofed that p38 MAPK mediates the expression of pro-inflammatory cytokines, such as TNF-α, IL-6 and IL-1β (Adhikary et al. 2004; Garbati et al. 2013). In the present research, the pro-inflammatory cytokines TNF-α, IL-6 and IL-1β in the myocardium tissue increased in the CHF group, and DOX-induced cardiac damage leads to an obvious increase in p38 MAPK activity. However, these inflammation responses were decreased and the activity of p38 MAPK was inhibited by SOJ treatment. These results showed that SOJ treatment protects against DOX-induced cardiac injury via inflammatory cytokines and p38 MAPK inhibition.

The present study has demonstrated that SOJ treatment improves cardiac function in DOX-induced CHF rats by increasing LVESP, LV ± dP/dtmax, FS and EF, and decreasing LVEDP, LVESD and LVEDD. This cardioprotective effect was further evidenced by the decreased levels of MDA, CK-MB, AST, LDH, TNF-α, IL-6 and IL-1β, inhibiting the p38 MAPK activation, and improving the activities of antioxidant enzymes. These results show that the cardioprotective effect of SOJ against CHF is mediated by various mechanisms, which include the inhibition of oxidative stress and suppression of inflammation cytokines.

It is well-know that saponins, especially steroidal saponins, are characteristic and representative bioactive components of *Ophiopogon japonicus.* These saponins show obvious activities, such as the protective effects on the cardiovascular system, anti-inflammation and antioxidation activities (Chen et al. 2016). In the present study, chemical characterization by HPLC-ELSD analysis demonstrated the presence of steroidal saponins in SOJ, including ophiopogonin B, ophiopogonin D and ophiopogonin D′, implying their contribution to the cardioprotective activity.

## Conclusions

SOJ treatment appears to possess an obvious cardioprotective effect in DOX-induced CHF. This is the first research to investigate this cardioprotective effect of SOJ via inhibiting the activity of p38 MAPK, suppressing the inflammatory cytokines, and alleviating the oxidative stress. In addition, our results clearly validate the traditional use of SOJ to treat CHF, and this research offers a basis for further clinical investigation of SOJ to protect against DOX-induced cardiac injury. Therefore, SOJ may represent a novel candidate to improve cardiac function. However, further study is still needed to demonstrate the underlying molecular mechanisms.
